# Supplementary material for: Integrating Non-spiking Interneurons in Spiking Neural Networks
Source: Front Neurosci. 2021 Mar 5;15:633945. doi: 10.3389/fnins.2021.633945 (PMC7973219; doi:10.3389/fnins.2021.633945)
Supplement: Supplementary file 1 [file Data_Sheet_1.PDF]

Supplementary Material

1 SUPPLEMENTARY TABLES AND FIGURES

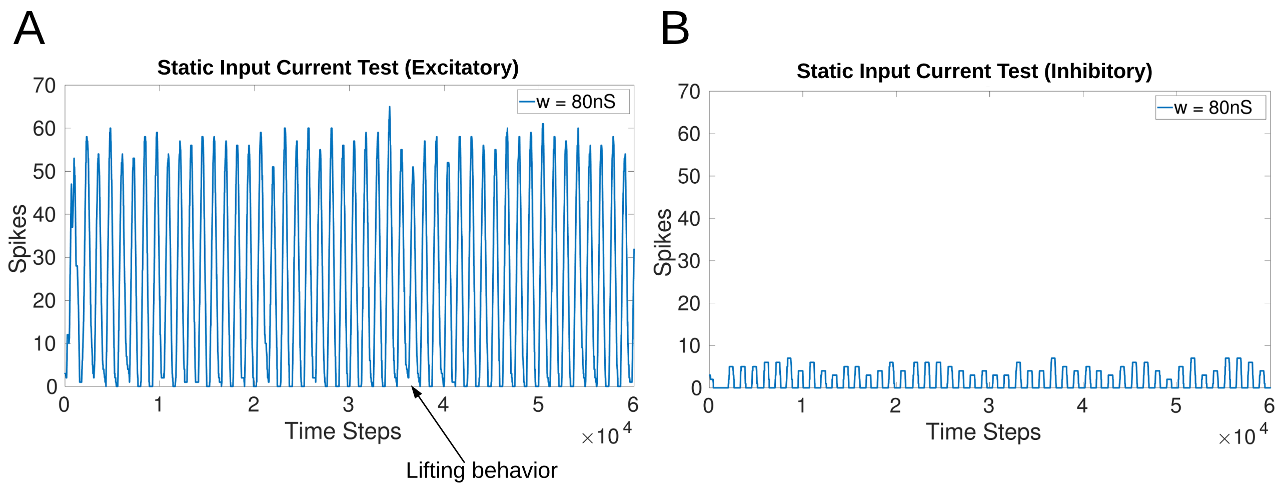

Figure S1. A and B: Results for a conductance weight of $w = 80nS$ and a constant input current to the NSI of $148pA$. A lifting behavior is noticeable within some time windows of the excitatory test (A) where the rate-coded output does not return to zero. The inhibitory test (B) output is initially suppressed.

Table S1. Start and end frequency per voltage characteristic manipulation test, only tests involving both sCPG neuron populations are shown. Tests for V_{th} and V_{reset} show frequency can be increased by a factor of 3. The V_m tests are inconclusive because they are unstable.

Frequency Comparison		
Test	Start Frequency (Hz)	End Frequency (Hz)
4_0 (V_{th} exc)	2.77	8.47
4_3 (V_{th} inh)	2.36	8.01
5_0 (V_{reset} exc)	2.94	8.94
5_3 (V_{reset} inh)	2.45	8.15
6_0 (V_m exc)	8.14	5.32
6_3 (V_m inh)	0.89	8.19

Table S2. Average maximum number of spikes based on the MNP output frequency. A static input current is provided to the NSI which changes the voltage threshold of the sCPG populations through an offset leading to different output frequencies.

Average Maximum Spikes based on MNP Output Frequency			
I_{input} (pA)	V_{th} (mV)	Average Freq (Hz)	Average Max Spikes
0.0	-55.6	3.0	96.6
29.6	-54.7	4.1	89.7
59.2	-53.7	5.2	70.0
88.8	-52.7	6.5	54.3
118.4	-51.7	7.5	51.5
148.0	-50.7	8.5	49.1

In all of the following plots, excitatory and inhibitory tests are compared to their respective counterparts. The vertical lines indicate when a change in input current occurs. The exact configuration details for each test are found in Tables 1 and 2 and visualized in Figures 4 and 5.

Tests Involving Both sCPG Neuron Populations

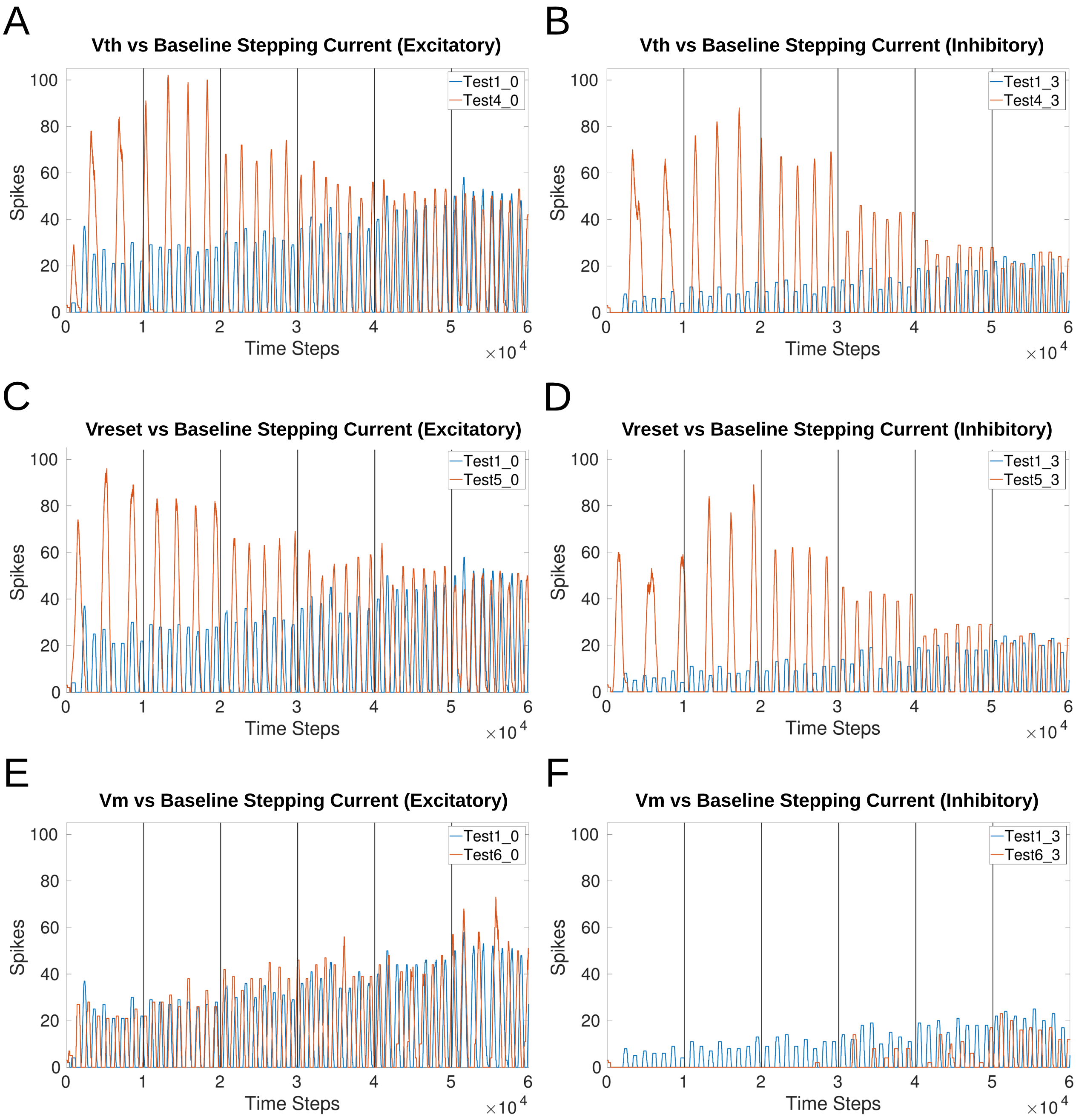

Figure S2. Comparison of voltage characteristic manipulation to the baseline MNP current injection step test. Outputs show that frequency is linearly affected by updating V_{th} and V_{reset} of sCPG neuron populations. Adding an offset to V_m of the post-synaptic neuron populations creates instability, the frequency of the signal does not increase linearly in comparison to the change in membrane potential. A and B: Voltage threshold is updated and current is injected to the MNP; C and D: Voltage reset value is updated and current is injected to the MNP; E:and F: Membrane potential is updated and current is injected to the MNP;

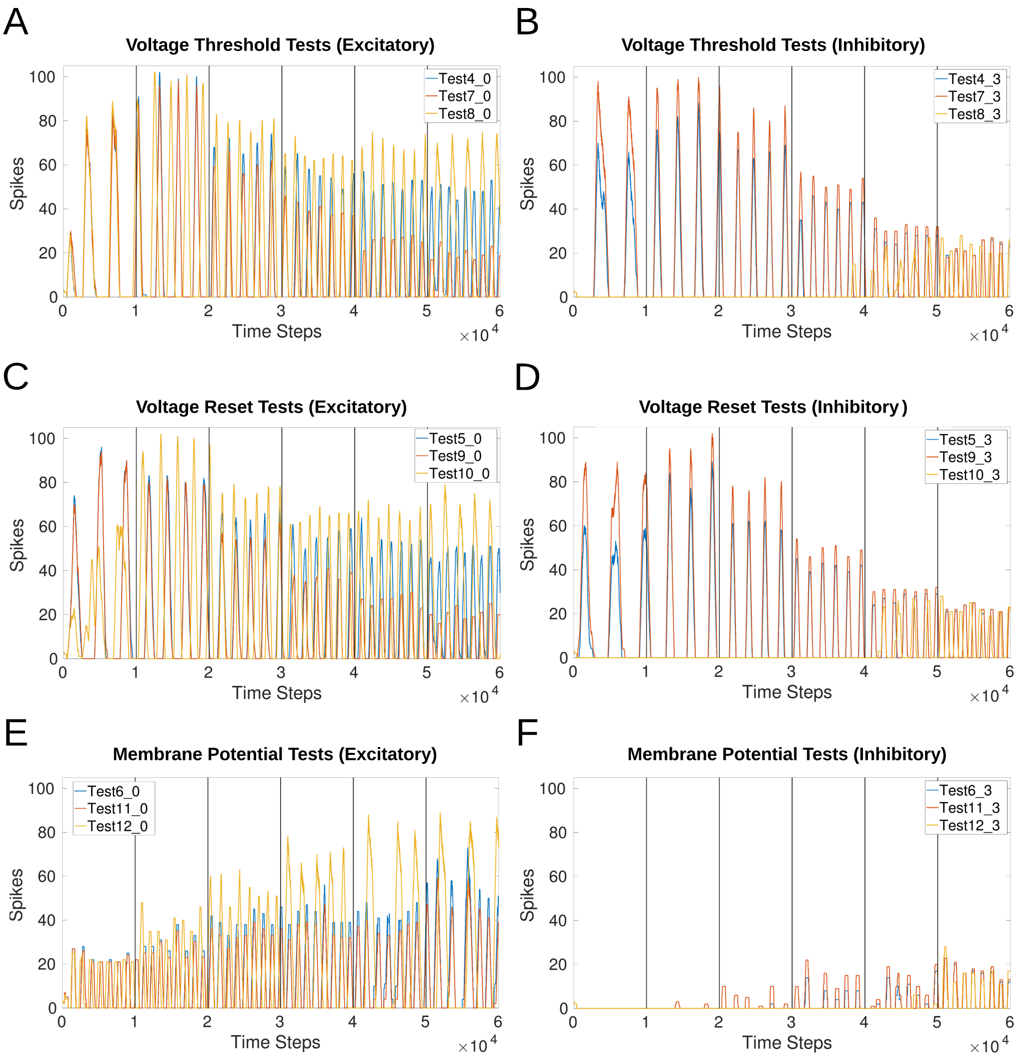

Figure S3. Comparison of voltage characteristic manipulation to the sCPG neuron populations with and without current injection to the sCPG neuron populations and MNP. Network behavior remains the same when current is injected to the MNP during frequency changes for all voltage characteristic manipulations. Current injection to the sCPG populations adds a phase shift and changes the output frequency. A and B: Voltage threshold is updated, Test 4 injects current to the MNP, Test 7 does not inject current to any post-synaptic populations, and Test 8 injects current to both the sCPG populations and the MNP; C and D: Voltage reset value is updated, Test 5 injects current to the MNP, Test 9 does not inject current to any post-synaptic populations, and Test 10 injects current to both the sCPG populations and the MNP; E and F: Membrane potential is updated, Test 6 injects current to the MNP, Test 11 does not inject current to any post-synaptic populations, and Test 12 injects current to both the sCPG populations and the MNP;

Tests Involving Single sCPG Neuron Populations

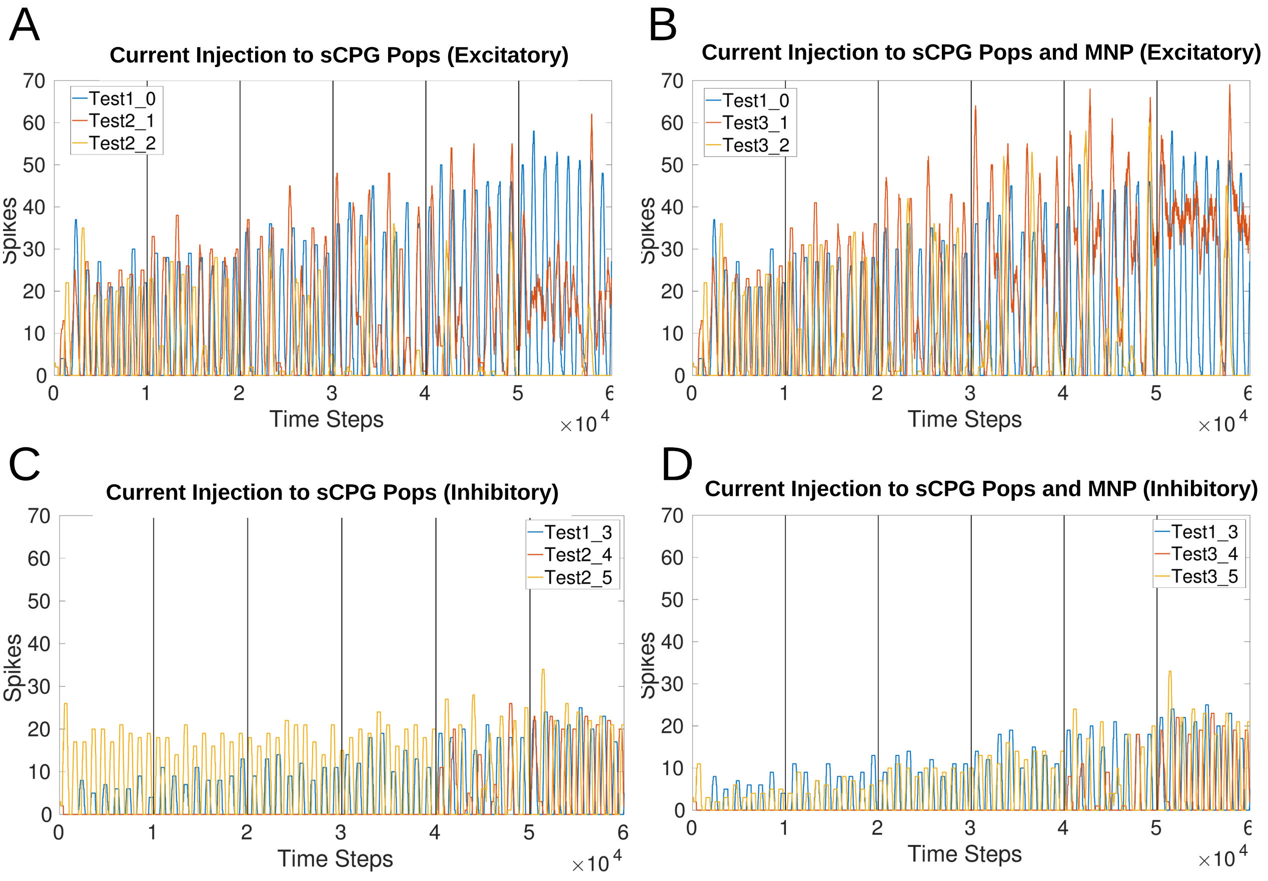

Figure S4. Comparison of current injections to either the excitatory (_1 and _4) or inhibitory (_2 and _5) sCPG neuron population to the baseline MNP current injection step test. Outputs show that current injection to a single sCPG population creates instability by over-inhibition suppressing output or over-excitation resulting in the signal not returning to zero. A and C: Current injection to the sCPG populations only.; B and D: Current injection to the sCPG populations and the MNP;

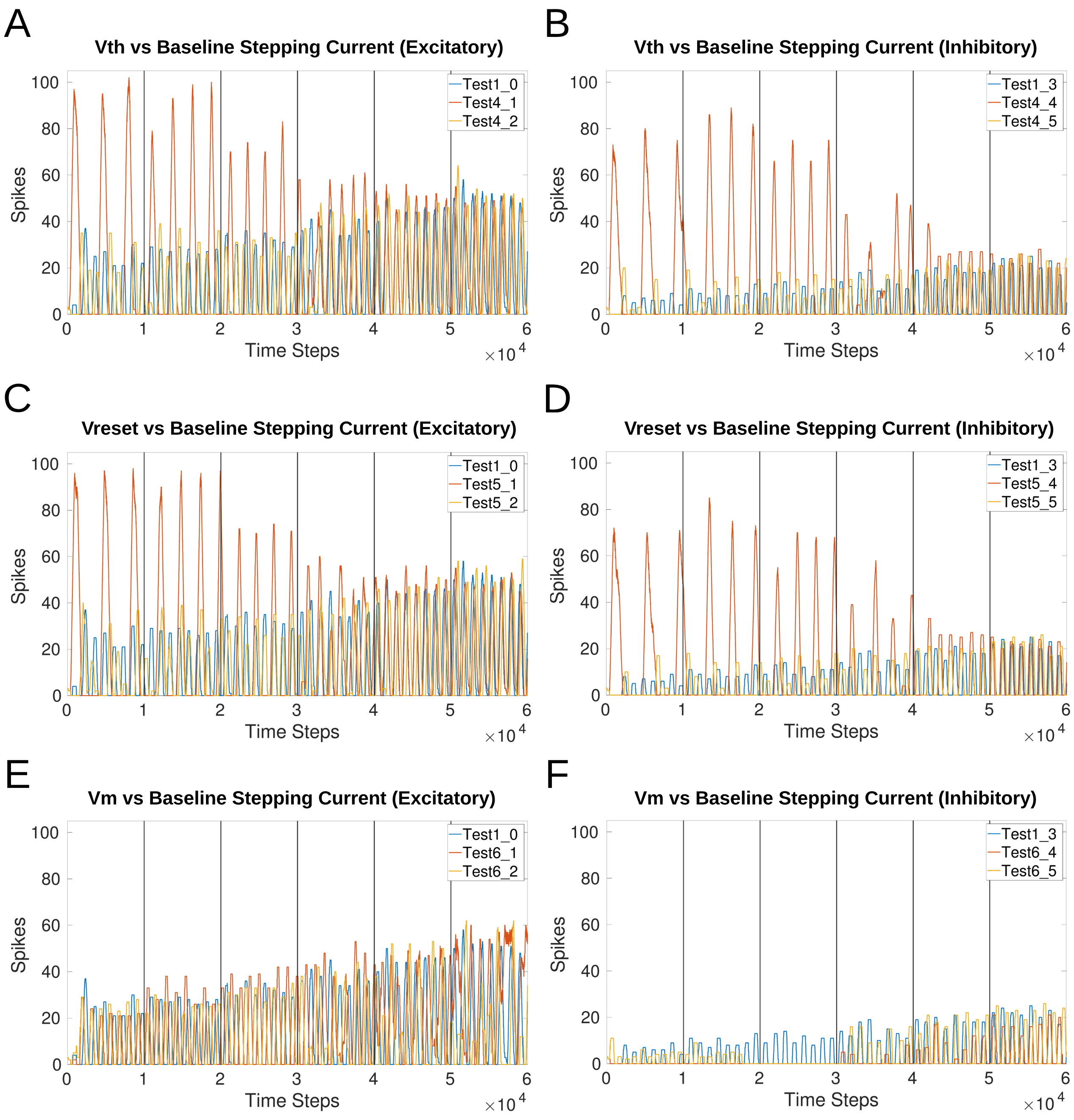

Figure S5. Comparison of voltage characteristic manipulation of either the excitatory (_1 and _4) or inhibitory (_2 and _5) sCPG neuron population to the baseline MNP current injection step test. Frequency is affected by updating V_{th} and V_{reset} of a single sCPG population but there are occasional ”double peaks”. Adding an offset to V_m of a single post-synaptic population changes the output characteristics of the signal. A and B: Voltage threshold is updated and current is injected to the MNP; C and D: Voltage reset value is updated and current is injected to the MNP; E and F: Membrane potential is updated and current is injected to the MNP;

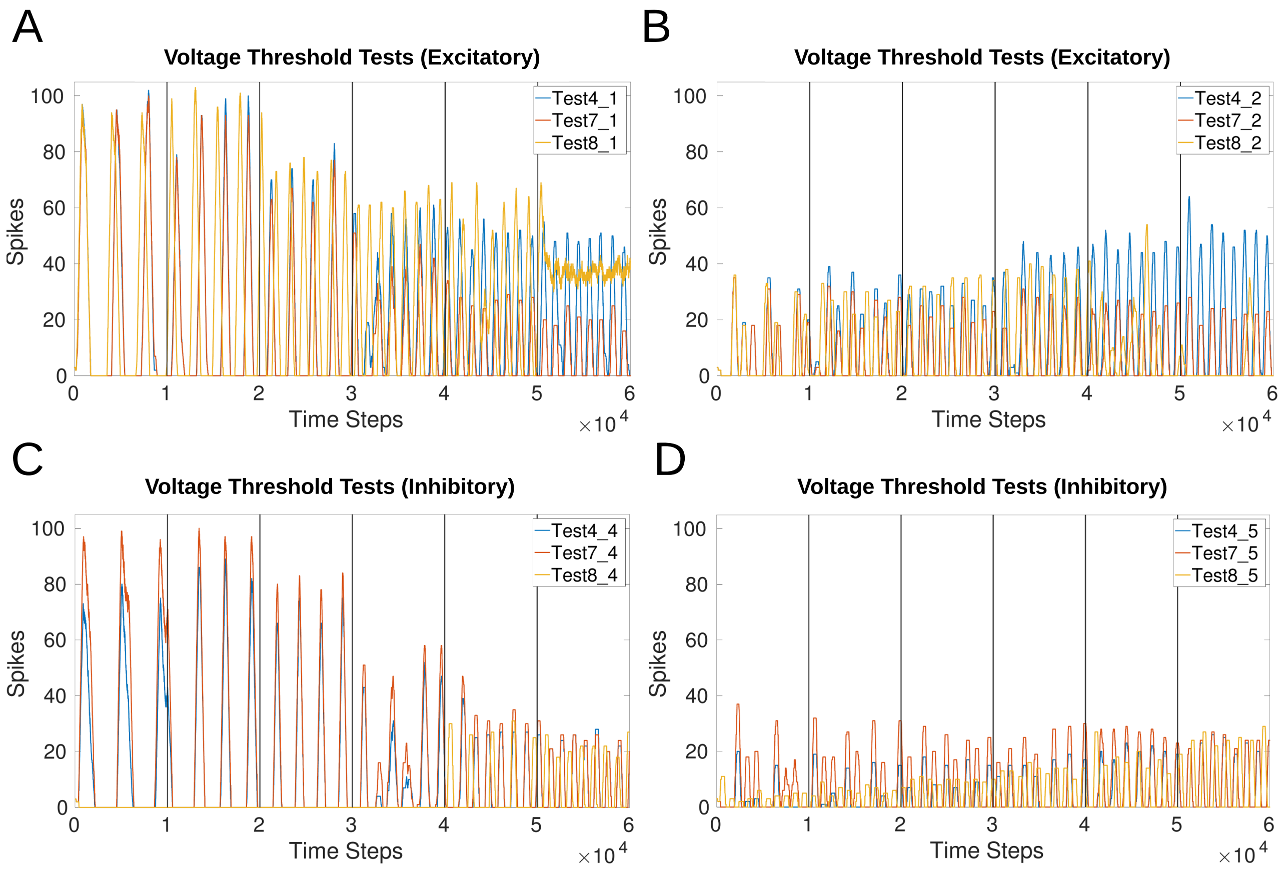

Figure S6. Comparison of V_{th} manipulation to either the excitatory (_1 and _4) or inhibitory (_2 and _5) sCPG neuron population. Test 4 injects current to the MNP, Test 7 does not inject current to any post-synaptic populations, and Test 8 injects current to both the sCPG populations and the MNP. A and B: Tests with excitatory current injection from the NSI; C and D: Tests with inhibitory current injection from the NSI;

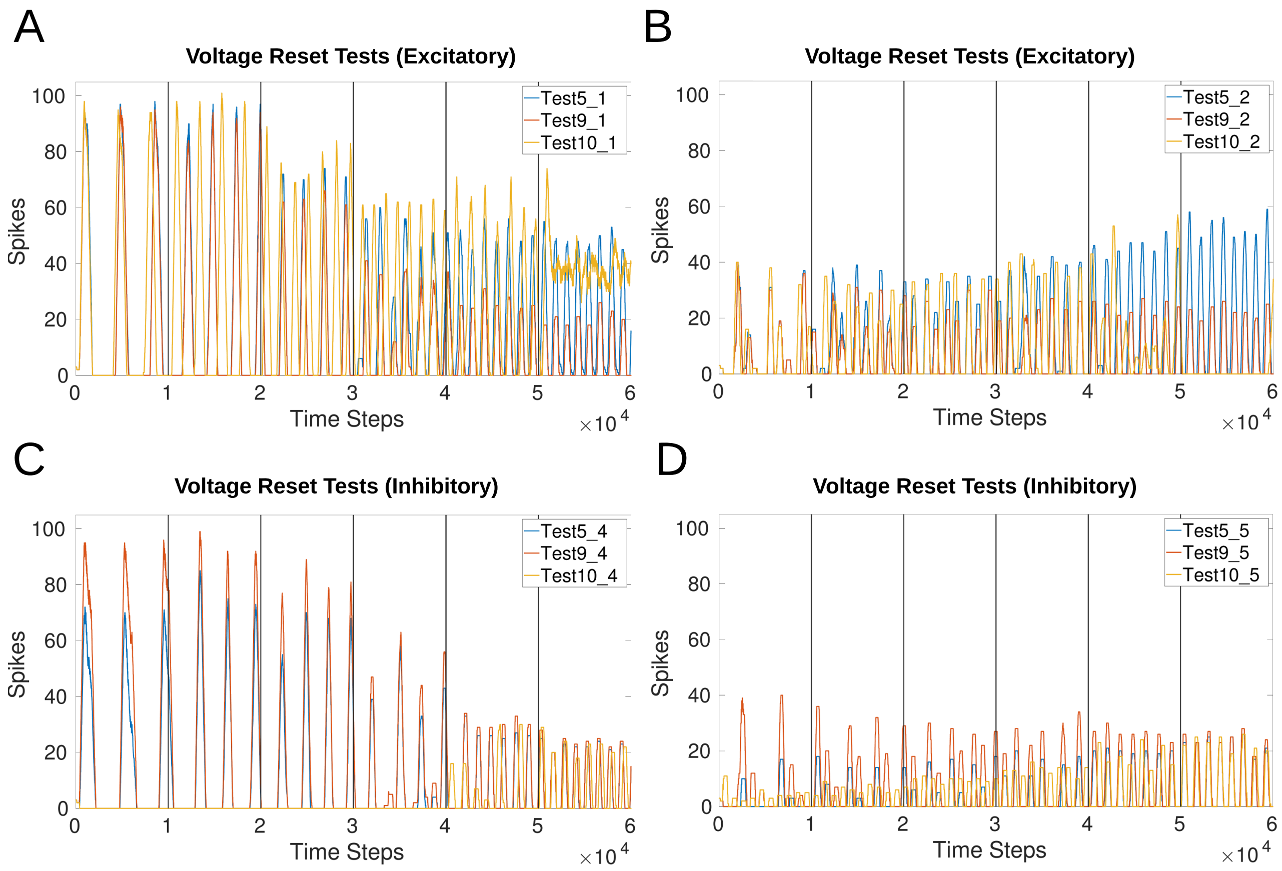

Figure S7. Comparison of V_{reset} manipulation to either the excitatory (_1 and _4) or inhibitory (_2 and _5) sCPG neuron population. Test 5 injects current to the MNP, Test 9 does not inject current to any post-synaptic populations, and Test 10 injects current to both the sCPG populations and the MNP. A and B: Tests with excitatory current injection from the NSI; C and D: Tests with inhibitory current injection from the NSI;

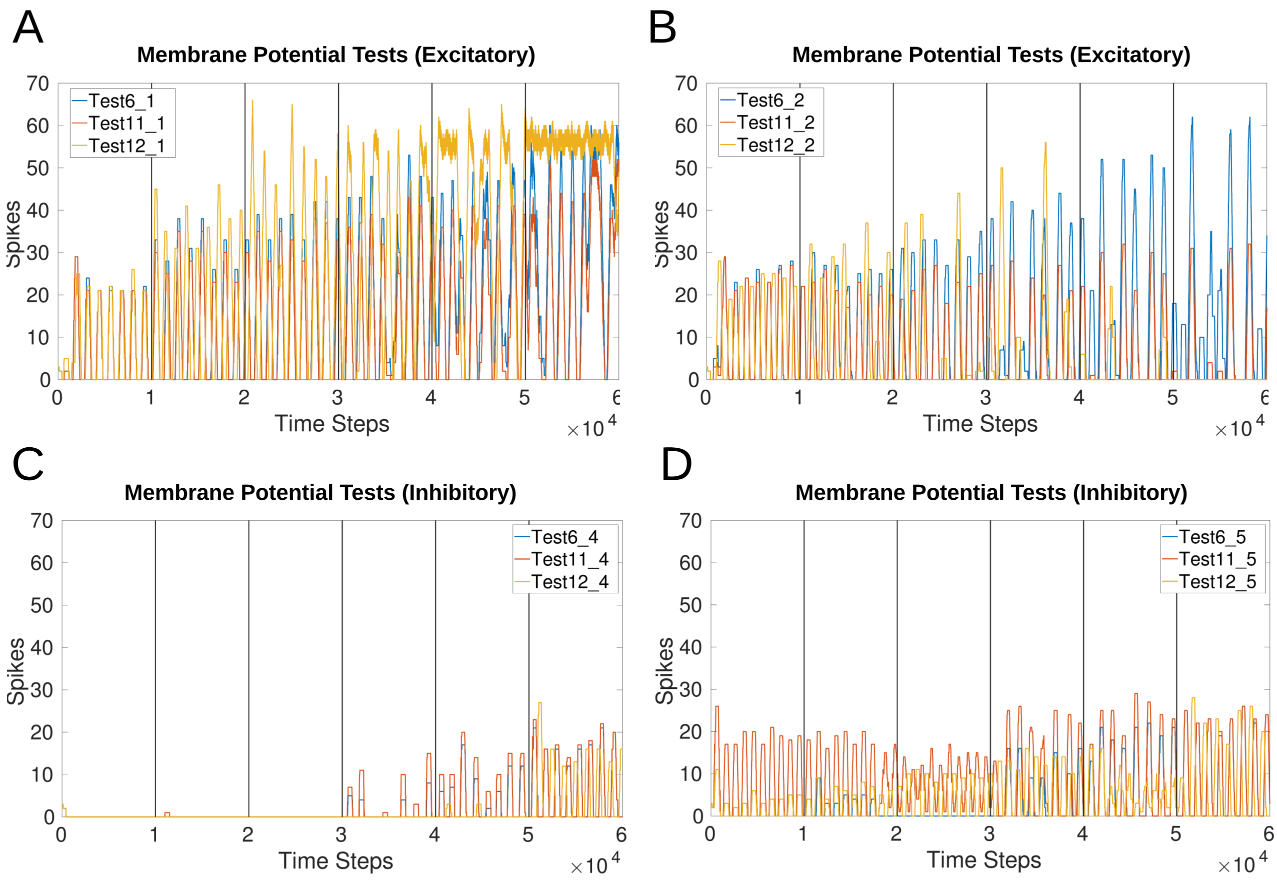

Figure S8. Comparison of V_m manipulation to either the excitatory (_1 and _4) or inhibitory (_2 and _5) sCPG neuron population. Test 6 injects current to the MNP, Test 11 does not inject current to any post-synaptic populations, and Test 12 injects current to both the sCPG populations and the MNP. A and B: Tests with excitatory current injection from the NSI; C and D: Tests with inhibitory current injection from the NSI;
